# Supplementary material for: Fluidigm2PURC: Automated processing and haplotype inference for double‐barcoded PCR amplicons
Source: Appl Plant Sci. 2018 Jun 28;6(6):e01156. doi: 10.1002/aps3.1156 (PMC6025812; doi:10.1002/aps3.1156)
Supplement: Supplementary file 1 [file APS3-6-e01156-s001.docx]

**APPENDIX S1.** Fluidigm2PURC example calculations and code.

**S1 HAPLOTYPE INFERENCE**

**S1.1 Inferring haplotypes with known ploidy**

To infer the maximum likelihood haplotype configurations with known ploidy, we use a multinomial likelihood that models the size of each cluster being considered as a haplotype. For an individual with ploidy level *K*, we take the first *K* clusters sorted by size and calculate the likelihood for a given partition as follows: each entry in a partition (*P*) contains the number of times that a particular haplotype is represented in the configuration. Given cluster sizes *C*_1_ through *C_K_*, and a sequencing error rate of ε, the log likelihood for a partition *P* is:

$$\mathcal{l}_{P}=\sum_{i=1}^{\left| P \right|} C_{i} \times\text{log}\left( \frac{P\left[ i \right]}{K} \right) + \sum_{j>\left| P \right|}^{K} C_{j} \times log(\varepsilon). (\text{S1.1})$$

Here $\left| P \right|$ represents the size of the partition.

***Example calculation***—To illustrate this calculation, let us consider a tetraploid individual that has the following sizes for the first four clusters: *C*_1_ = 285, *C*_2_ = 95, *C*_3_ = 10, and *C*_4_ = 8. We will also use an error rate of ε = 0.002. Table S1.1 has the list of possible haplotype configurations and their corresponding log-likelihood values. The maximum likelihood haplotype configuration has three copies of haplotype 1 and one copy of haplotype 2. The explicit likelihood calculation for this haplotype configuration proceeds as follows:

$$\mathcal{l}_{\left( 3,1 \right)}= 285 \times\log\left( \frac{3}{4} \right)+95 \times\log\left( \frac{1}{4} \right)+10 \times\log\left( 0.002 \right)+ 8 \times\log\left( 0.002 \right)= -325.5503.$$

(S1.2)

**Table S1.1.** Haplotype configurations and their corresponding log-likelihoods for a tetraploid with ordered cluster sizes equal to 285, 95, 10, and eight. The haplotype configuration with three copies of haplotype 1 and one copy of haplotype 2 has the highest likelihood (boldfaced).

| **Haplotype configuration** | **Log-likelihood** |
| --- | --- |
| (4,0,0,0) | −702.2507 |
| **(3,1,0,0)** | −**325.5503*** |
| (2,2,0,0) | −375.2589 |
| (2,1,1,0) | −392.8247 |
| (1,1,1,1) | −551.7452 |

**S1.2 Inferring haplotypes with unknown ploidy**

Inferring haplotype configurations for individuals with unknown ploidy levels involves distinguishing clusters that are likely to be “real” haplotypes from those that are likely to be errors. We do this by considering a set of models that range from treating all clusters as errors, to one where all clusters are real haplotypes. The models in between successively treat each cluster in the ordered set as a real haplotype (clusters are sorted by size). For an individual with *N* clusters, there are *N* + 1 models to test. Each of these models has *H* real haplotypes (0,…,*H*) and *N*-*H* errors (*H+*1,…,*N*). The likelihood for each of these models is the sum of the cluster sizes (*C*_1_,…,*C*_N_) multiplied by the probability that they are sequencing errors (ε) or not (1− ε). The log-likelihood for a model with *H* haplotypes is given by:

$$\mathcal{l}_{H}= \sum_{i=1}^{H} C_{i} \times\log(1-\varepsilon)+ \sum_{j>H}^{N} C_{j} \times\log(\varepsilon). (\text{S1.2})$$

To determine the most likely haplotype configuration, we calculate how much the likelihood increases over the previous model when another haplotype is added (the likelihood is monotonically increasing). We also normalize these differences by the total change in likelihood from the model with *H* = 0 to the model with *H* = *N*. If this value is less than a given cutoff (we use a default of 0.10), the previous model is treated as the best configuration. Because the cluster sizes are ordered, the increase in the log-likelihood will always be smaller for any additional haplotypes.

***Example calculation***—We will illustrate this procedure using an example for six clusters with the following sizes: *C*_1_ = 425, *C*_2_ = 210, *C*_3_ = 145, *C*_4_ = 18, *C*_5_ = 11, *C*_6_ = 7. Using an error rate of 0.002, the R code below will calculate the likelihoods for the different models as well as the relative increase for each of them (Table S1.2).

R code:

ullik_0 <- 425*log(0.002) + 210*log(0.002) + 145*log(0.002)

+ 18*log(0.002) + 11*log(0.002) + 7*log(0.002)

ullik_1 <- 425*log(1-0.002) + 210*log(0.002) + 145*log(0.002)

+ 18*log(0.002) + 11*log(0.002) + 7*log(0.002)

ullik_2 <- 425*log(1-0.002) + 210*log(1-0.002) + 145*log(0.002)

+ 18*log(0.002) + 11*log(0.002) + 7*log(0.002)

ullik_3 <- 425*log(1-0.002) + 210*log(1-0.002) + 145*log(1-0.002)

+ 18*log(0.002) + 11*log(0.002) + 7*log(0.002)

ullik_4 <- 425*log(1-0.002) + 210*log(1-0.002) + 145*log(1-0.002)

+ 18*log(1-0.002) + 11*log(0.002) + 7*log(0.002)

ullik_5 <- 425*log(1-0.002) + 210*log(1-0.002) + 145*log(1-0.002)

+ 18*log(1-0.002) + 11*log(1-0.002) + 7*log(0.002)

ullik_6 <- 425*log(1-0.002) + 210*log(1-0.002) + 145*log(1-0.002)

+ 18*log(1-0.002) + 11*log(1-0.002) + 7*log(1-0.002)

total_diff <- abs(ullik_0 - ullik_6)

diff_1 <- ullik_1 - ullik_0; rel_diff_1 <- diff_1 / total_diff

diff_2 <- ullik_2 - ullik_1; rel_diff_2 <- diff_2 / total_diff

diff_3 <- ullik_3 - ullik_2; rel_diff_3 <- diff_3 / total_diff

diff_4 <- ullik_4 - ullik_3; rel_diff_4 <- diff_4 / total_diff

diff_5 <- ullik_5 - ullik_4; rel_diff_5 <- diff_5 / total_diff

diff_6 <- ullik_6 - ullik_5; rel_diff_6 <- diff_6 / total_diff

Using a cutoff of 0.10, we can see that the configuration (1,1,1,0,0,0) is the last haplotype configuration that increases the likelihood by more than 10%, meaning that the most likely scenario is that the first three clusters are real haplotypes and the last three are errors.

**Table S1.2.** Haplotype configurations for an individual with six clusters. The ordered cluster sizes are 425, 210, 145, 18, 11, and seven. A model where the first three clusters are real haplotypes is the best fit (boldfaced). The last three haplotype configurations did not meet the threshold and are marked in red.

| **Haplotype configuration** | **Log-likelihood** | **Relative increase** |
| --- | --- | --- |
| (0,0,0,0,0,0) | −5071.12 | NA |
| (1,0,0,0,0,0) | −2430.763 | 0.5208 |
| (1,1,0,0,0,0) | −1126.115 | 0.2574 |
| **(1,1,1,0,0,0)** | −**225.2875** | **0.1777*** |
| (1,1,1,1,0,0) | −113.4605 | 0.0221 |
| (1,1,1,1,1,0) | −45.12188 | 0.0135 |
| (1,1,1,1,1,1) | −1.633634 | 0.0086 |

**S2 EXAMPLE ANALYSIS**

We have provided the sequence data from the *PIS_3* and *PIS_4* loci for the six species of *Thalictrum* in the files 'Thalictrum_R1.fastq.gz' and 'Thalictrum_R2.fastq.gz'. The following sections will walk through the analyses that we did to compare haplotypes inferred using Fluidigm2PURC and dbcAmplicons (Uribe-Convers et al., 2016). To install all of the necessary software, please follow the instructions on the ReadTheDocs page for Fluidigm2PURC: <http://fluidigm2purc.readthedocs.io/>.

**S2.1 Fluidigm2PURC**

To analyze the data using Fluidigm2PURC, we first run the *fluidigm2purc* script:

$ fluidigm2purc -f Thalictrum -o thalictrum-f2p -j 2

All of the reads coming from each locus will be written to separate FASTA files that are put into the directory thalictrum-f2p-FASTA/ (the -o option gives the output prefix). Next, we change into this directory and run PURC (Rothfels et al., 2017) on the *PIS_3* and *PIS_4* loci separately.

$ cd thalictrum-f2p-FASTA/

$ purc_recluster.py -f PIS_3.fasta -o PIS_3 -c 0.997 0.995 0.99 0.997 -s 2 5 --clean

$ purc_recluster.py -f PIS_4.fasta -o PIS_4 -c 0.997 0.995 0.99 0.997 -s 2 5 --clean

Here the results are written to separate directories: PIS_3/ and PIS_4/. We will work on these loci in their respective directories by running the *crunch_clusters* script. For analyses 1 and 2, we do not change the taxon table to include ploidy information. However, before running step 3, we add the ploidy levels of the taxa sampled. Running the first two cluster crunching steps for both loci before adding the ploidy information is best so that we do not have to change the taxon table more than once. We also manually renamed the output FASTA files after each *crunch_clusters* run so that the files do not get overwritten. In a normal situation, you would not need to run all three of these analyses unless you wanted to compare the differences between assuming ploidy levels are known or unknown.

## Working on the PIS_3 locus

# 1. Getting consensus loci using the --haploid flag

$ cd PIS_3/

$ crunch_clusters -i PIS_3_clustered_reconsensus.afa -l PIS_3 \

-s ../../thalictrum-f2p-taxon-table.txt -e ../../thalictrum-f2p-locus-err.txt \

--realign --clean 0.33 --haploid

# 2. Assuming we don’t know ploidy levels

$ crunch_clusters -i PIS_3_clustered_reconsensus.afa -l PIS_3 \

-s ../../thalictrum-f2p-taxon-table.txt -e ../../thalictrum-f2p-locus-err.txt \

--realign --clean 0.33

# 3. Getting unique haplotypes with known ploidy information (add ploidy info first)

$ crunch_clusters -i PIS_3_clustered_reconsensus.afa -l PIS_3 \

-s ../../thalictrum-f2p-taxon-table.txt -e ../../thalictrum-f2p-locus-err.txt \

--realign --clean 0.33 --unique_haps

## Working on the PIS_4 locus

# 1. Getting consensus loci using the --haploid flag

$ cd ../PIS_4/

$ crunch_clusters -i PIS_4_clustered_reconsensus.afa -l PIS_4 \

-s ../../thalictrum-f2p-taxon-table.txt -e ../../thalictrum-f2p-locus-err.txt \

--realign --clean 0.33 --haploid

# 2. Assuming we don’t know ploidy levels

$ crunch_clusters -i PIS_4_clustered_reconsensus.afa -l PIS_4 \

-s ../../thalictrum-f2p-taxon-table.txt -e ../../thalictrum-f2p-locus-err.txt \

--realign --clean 0.33

# 3. Getting unique haplotypes with known ploidy information (add ploidy info first)

$ crunch_clusters -i PIS_4_clustered_reconsensus.afa -l PIS_4 \

-s ../../thalictrum-f2p-taxon-table.txt -e ../../thalictrum-f2p-locus-err.txt \

--realign --clean 0.33 --unique_haps

**S2.2 dbcAmplicons (*reduce_amplicons.R*)**

To get haplotypes with dbcAmplicons, we first run the *reduce_amplicons.R* script. We trimmed 20 bases from read 1 and 40 bases from read 2. We also run both consensus- and occurrence-based haplotype inference using the -p option. The output directory is specified using the -o option.

$ reduce_amplicons.R -p consensus,occurrence --trim-1 20 --trim-2 40 \

-o thalictrum-dbc Thalictrum

Next, we need to align the output of the *reduce_amplicons.R* script for the consensus- and occurrence-based haplotype inference methods. We first change into the consensus.split_amplicon/ directory in the main thalictrum-dbc/ output directory and then align the haplotypes for *PIS_3* and *PIS_4* using MAFFT (Katoh et al., 2013).

$ cd thalictrum-dbc/consensus.split_amplicon/

$ mafft --auto --quiet Amplicon.PIS_3.merged.fasta > PIS_3-consensus.fasta

$ mafft --auto --quiet Amplicon.PIS_4.merged.fasta > PIS_4-consensus.fasta

Next, we change back into the main output directory and then change into the occurrence.split_amplicon/ directory to align the occurrence-based haplotypes inferred by dbcAmplicons.

$ cd ../occurrence.split_amplicon/

$ mafft --auto --quiet Amplicon.PIS_3.merged.fasta > PIS_3-occurrence.fasta

$ mafft --auto --quiet Amplicon.PIS_4.merged.fasta > PIS_4-occurrence.fasta

All of the resulting haplotype files were then read into Geneious to visualize and calculate alignment statistics (Kearse et al., 2012). Parsimony informative sites were calculated in MEGA7 (Kumar et al., 2016).

**SUPPLEMENTAL LITERATURE CITED**

Katoh, S. 2013. MAFFT multiple sequence alignment software version 7: Improvements in performance and usability. *Molecular Biology and Evolution* 30: 772–780.

Kearse, M., R. Moir, A. Wilson, S. Stones-Havas, M. Cheung, S. Sturrock, S. Buxton, et al. 2012. Geneious Basic: An integrated and extendable desktop software platform for the organization and analysis of sequence data. *Bioinformatics* 28: 1647–1649.

Kumar, S., G. Stecher, and K. Tamura. 2016. MEGA7: Molecular Evolutionary Genetics Analysis version 7.0 for bigger datasets. *Molecular* *Biology and Evolution* 33: 1870–1874.

Rothfels, C. J., F.-W. Li, and K. M. Pryer. 2017. Next-generation polyploid phylogenetics: Rapid resolution of hybrid polyploid complexes using PacBio single-molecule sequencing. *New Phytologist* 213: 413–429.

Uribe-Convers, S., M. L. Settles, and D. C. Tank. 2016. A phylogenomic approach based on PCR target enrichment and high throughput sequencing: Resolving the diversity within the South American species of *Bartsia* L. (Orobanchaceae). *PLoS ONE* 11: e0148203.
